# Social and contextual factors associated with drinking before, during and after watching Australian Football League games: A pilot ecological momentary assessment study

**DOI:** 10.1111/dar.13706

**Published:** 2023-07-03

**Authors:** Amy Pennay, Kelly van Egmond, Dan Anderson‐Luxford, Cassandra J. C. Wright, Gabriel Caluzzi, Michael Livingston, Geoff Dickson, Matthew Nicholson, Emmanuel Kuntsche

**Affiliations:** ^1^ Centre for Alcohol Policy Research La Trobe University Melbourne Australia; ^2^ Burnet Institute Melbourne Australia; ^3^ Menzies School of Health Research Darwin Australia; ^4^ National Drug Research Institute, Curtin University Perth Australia; ^5^ Department of Management and Marketing La Trobe University Melbourne Australia; ^6^ Monash Malaysia Kuala Lumpur Malaysia; ^7^ Centre for Sport and Social Impact, La Trobe University Melbourne Australia; ^8^ Institute of Psychology, Eötvös Loránd University Budapest Hungary

**Keywords:** alcohol, Australian Football League, ecological momentary assessment, heavy drinking, sport

## Abstract

**Introduction:**

The aim of this study was to: (i) determine the feasibility of using ecological momentary assessment to collect data from Australian Football League (AFL) fans; (ii) explore pre‐game, during‐game and post‐game consumption patterns of AFL fans; and (iii) explore the social and setting‐related factors associated with risky single occasion drinking (5+ drinks) among AFL fans.

**Methods:**

Thirty‐four participants completed up to 10 ecological momentary assessment surveys before, during and after 63 AFL games (*n* = 437 completed surveys). Surveys collected data about their drinking, and their social and environmental milieu (e.g., location, company). Binary logistic regression analyses clustered by participant identified which game‐day characteristics were associated with higher odds of risky single occasion drinking. Significant differences between pre‐game, during‐game and post‐game drinking on social and environmental factors were explored using pairwise comparisons.

**Results:**

Risky single occasion drinking was more likely when games began in the early‐afternoon (1–3 pm) than late‐afternoon (3–6 pm), when participants watched the game at a stadium or pub compared to home, and when participants watched the game with friends compared to family. Pre‐drinking was more likely before night games and post‐drinking was more likely after day games. Drinking during the game was heavier when watching the game at a pub and when watching with a combined group of friends and family.

**Discussion and Conclusions:**

Preliminary findings suggest that social and contextual factors matter in the way alcohol is consumed while watching AFL games. These findings require further investigation in larger samples.

## 1 INTRODUCTION

Research shows that some social and settings‐based subcultures drink more heavily than others, suggesting that intervening in heavy drinking subcultures might be a useful complement to population‐level alcohol control policies [1, 2]. One internationally recognised heavy drinking subculture is sports fans. A large American study identified that compared to non‐sport fans, sport fans consumed more alcohol and were more likely to engage in heavy episodic drinking and report alcohol‐related problems [3]. American and Swedish studies identified that more than 40% of sports fans at football and baseball games had a blood alcohol concentration (BAC) greater than 0.08% [4, 5].

In Australia, the most popular sport among viewers is Australian Rules Football, which is governed by the Australian Football League (AFL) [6]. High rates of alcohol‐related ambulance and emergency department attendances are recorded after professional AFL games [7], underscoring the importance of preventing heavy drinking among AFL fans. However, there has been little research on the drinking practices of this group. One Australian study identified that sports bar patrons consumed an average of six standard drinks at sports bars [8], but little else is known about drinking and spectatorship of professional sport in Australia. Further information is important to know how contextual factors influence how much, and in what ways, AFL spectators are consuming alcohol to meaningfully inform policy and prevention strategies.

Event‐level studies suggest that a range of factors might contribute to heavy drinking during an event (e.g., mood, day of the week, location and drinking group characteristics) [9]. For example, in studies with young people in nightlife environments in Switzerland, event‐level factors (e.g., friends present in a situation) had a strong effect on drinking levels [10, 11]. Among sports fans, higher BAC levels were evident in American baseball and football spectators who were younger age, attended Monday or Friday (rather than Sunday) games, were breathalysed later at night and engaged in pre‐drinking [4]. However, these studies collected data after the game (potentially before the end of the drinking session) and their applicability in other contexts is unclear. For example, in Australia, 10 of the 18 AFL teams are in the state of Victoria. This means marquee matches between two Melbourne‐based teams can attract more than 100,000 spectators, but crowds can also be very small when non‐Victorian teams travel to Victoria. Games are mostly played on the weekend, sometimes on Thursday, and rarely on Mondays, but more popular teams are likely to be scheduled on Friday night or Saturday night for television ratings purposes. Further, there are international differences in drinking practices, with no culture of tailgating—the American practice of drinking in parking lots before the game—in Australia, though pre‐drinking at home or a pub is still common.

Previous studies examining event‐level drinking during sporting events have tended to rely on breathalysers (e.g., [4, 5]). Breathalysers are a useful but resource‐intensive way of collecting information at a single time point. Even when BAC data are collected at multiple time points, pre‐ and post‐event patterns of consumption tend to remain unknown (e.g., [5]). This is important, because effective prevention and regulatory strategies rely on an understanding of whether patrons become intoxicated before, during or after the event. Ecological momentary assessment (EMA) techniques, which involve the completion of repeated‐measures surveys typically collected on mobile phones [12], are useful for recording drinking practices and other factors that occur over longer periods of time. EMA techniques can also capture nuanced changes during a short time frame [13], such as between leaving a sporting event and attending a post‐game venue. This suggests EMA is a promising method for investigating the contextual factors impacting consumption patterns over the duration of a sporting event. However, it is unknown if an EMA approach is feasible in sports settings where spectators are preoccupied with entertainment beyond or different to that in nightlife settings (where most EMA studies on alcohol have been undertaken). It also not known the best way to recruit participants for an in‐situ study on sports spectatorship and alcohol.

The aim of this pilot study was to:

- Determine the feasibility of EMA to collect alcohol consumption data from AFL spectators (in terms of recruitment, participant engagement and data interpretability);
- Explore pre‐game, during‐game and post‐game consumption patterns of AFL fans; and
- Explore the social and setting‐related factors associated with heavy drinking among AFL fans.

## 2 METHODS

### 2.1 Participants and recruitment

Participants were initially recruited through a street intercept approach at Melbourne's largest sport stadium at four AFL games over two weekends. Teams of 10 researchers approached participants and asked them to complete a screening questionnaire. Participants who completed a screening questionnaire were eligible to win an AUD 200 voucher. Those who were eligible for participation were contacted a few days later by email or phone to explain the EMA component, which would involve completing surveys during three AFL games over three consecutive weekends. This approach yielded a low engagement rate and consequently recruitment was enhanced through paid targeted social media advertising and social media promotion on the research team's personal and professional social media accounts (i.e., Twitter, Facebook, Instagram). The social media advertisement directed participants through to the screening questionnaire.

Our pilot sample size aim was to collect data for 60 matches. Our final sample consisted of 34 participants who provided data for 63 AFL games in total (range 1–4 games per person; not all participants completed the EMA surveys for three consecutive games, and so a fourth round was offered to interested participants). Study inclusion criteria were: ≥18 years; recent consumer of alcohol while watching AFL (i.e., ≥2 drinks while watching AFL in the preceding month); watching their AFL team at least fortnightly; willingness to use their smartphone for the study; and able to read and understand English. Exclusion criteria were being pregnant or breastfeeding. Written consent was collected from participants. The study was approved by the Human Research Ethics Committee at La Trobe University (HEC18524).

### 2.2 Procedure

Data were collected across four consecutive weeks in August and September 2019. Participants were asked to download the RealLife Exp app to their smartphones (www.lifedatacorp.com). There are nine AFL games each week between the Australian winter months of March and October, usually played on Friday night, Saturday day, late afternoon or night, and Sunday day and late afternoon (Thursday night matches have become more common in the years after the study, but did not feature during our data collection period). Day games start between 1 and 3 pm, late afternoon games between 3 and 6 pm and night games between 7 and 8.30 pm. Games last for approximately 2.5 h. AFL games are divided into four quarters of approximately 30 min each.

Each week on Thursday, participants were sent an EMA survey asking them to select a game they would be watching (multiple participants could theoretically select the same match; participants could only select one match per week). This answer triggered their game‐day EMA surveys, timed to their game's scheduling. If participants did not respond to this survey, they did not receive any match day surveys. In total, out of 72 ‘trigger’ surveys administered, 9 were missed (11.11%) and thus, no match‐day surveys were sent for these events.

Participants who completed the trigger survey received notifications on their smartphone that an EMA survey was ready for completion at the following intervals: 10 min before the game, at quarter‐time, half‐time, three‐quarter‐time, after the game, every 2 h until midnight and then one final next‐day survey (a maximum of 10 surveys). On game day, participants continued to receive notifications if they missed the prior survey. Of a total of 511 game day and next‐day surveys sent to participants, 437 were completed and 74 (14.5%) were missed/not completed.

### 2.3 Measures

Sex, age, drinking habits and the alcohol use disorders identification test (a screening test for heavy drinking [14]) were assessed in the screening questionnaire.

On game day, to measure alcohol consumption, the pre‐game survey asked participants how many drinks they had consumed until then. Subsequent surveys asked how many drinks they had consumed since their last completed survey. This was deliberately phrased this way so that if a survey was missed, participants would report the number of drinks consumed since their last completed survey (i.e., not just their drinks in the previous quarter of the game). Participants were shown an image which displayed pictures of beverages accompanied by the number of standard drinks (10 g of ethanol) in each beverage.

Location during and after the game was assessed with the response options: home, others' home, stadium, pub, other. To ascertain company, we asked who they were with at each time point (e.g., family, friends, other—a combination could be selected). Participants were asked how many people they were watching the game with that they knew (i.e., not how many were at the pub or stadium), how many of those were men and how many were under the age of 30. Prior to the game participants were asked if they expected their team to win and after the game whether their team had won the match.

### 2.4 Analysis

Data were imported into Stata version 14 [15]. Descriptive statistics were produced for demographics and match day information. Our primary outcome measure was risky single occasion drinking (RSOD), defined as five or more standard drinks by Australia's National Health and Medical Research Council [16]. This was calculated by summing up the total drinks (pre, during and after the game) reported by participants on one match day. Binary logistic regression analyses (clustered by participant) identified which individual and match‐day characteristics were associated with RSOD. Drinking trajectories (mean drinks at three intervals: pre‐game, during‐game and post‐game) were explored by the social and environmental characteristics significant in the regression models (e.g., match time, location of viewing and company). Pairwise comparisons (Tukey's range test), with degrees of freedom and Cohen's *d* as a measure effect size, were run to detect significant differences between pre‐game, during‐game and post‐game drinking on these social and environmental factors.

## 3 RESULTS

Table 1 displays the characteristics of the 34 participants and the 63 games for which data were collected (which comprised 24 unique matches). For the 63 games, participants were most likely to watch at their own home (42%) or the stadium (30%). They were equally likely to attend games on Friday, Saturday or Sunday, but more likely to spectate night‐time games (43%) and late afternoon games (36%). They were most likely to watch the game with friends (51%) or family (30%), and approximately one‐third (35%) went to a location other than home after the game. Most (84%) of the sample consumed alcohol during the game, with approximately a third drinking before and after the game. Mean total drinks for the day was 6.0 standard drinks, with most of this (3.6) occurring during game time. Just over one‐third (34.9%) of events were RSOD episodes.

**TABLE 1:** Sample characteristics and results of binary logistic regression analyses (clustered by participant) predicting risky single occasion drinking (5+ drinks).

	Sample characteristics	Binary logistic regression 5+ drinks (34.9% events)
		ORs (CI)
**Individuals (*n* = 34)**		
Women^a^	11 (33.3%)	
Mean age	28.3 (18–62; SD 11.3)	
Mean AUDIT‐C	6.0 (3–9; SD 1.6)	
**Games (*n* = 63)**		
Any drinking	50 (89%)	
Pre‐game drinking	24 (38%)	
During‐game drinking	53 (84%)	
Post‐game drinking	21 (33%)	
Mean no. drinks (those who consumed any)		
Pre‐game	0.9 (0–6; SD 1.5)	
During game	3.6 (0–12; SD 3.1)	
Post‐game	1.4 (0–18; SD 3.6)	
Total	6.0 (1–29; SD 5.9)	
Day of game		
Friday	19 (30%)	Ref
Saturday	23 (37%)	1.32 (0.34, 5.11)
Sunday	21 (33%)	0.54 (0.12, 2.33)
Start time of game		
Day (1–3 pm)	13 (21%)	Ref
Late afternoon (3–6 pm)	23 (36%)	0.25 (0.07, 0.88)*
Night (6 pm or later)	27 (43%)	0.93 (0.24, 3.49)
Expect team to win	35 (62%)	1.04 (0.31, 3.52)
Team won the game	27 (48%)	1.38 (0.46, 4.12)
Location of watching game		
Home	26 (42%)	Ref
Stadium	18 (30%)	5.5 (1.33, 22.81)*
Pub	6 (10%)	27.5 (2.29, 330.01)*
Combination of home/stadium/pub	5 (8%)	1.4 (0.11, 17.82)
Someone else's home	6 (10%)	5.5 (0.68, 44.68)
Location after the game		
Home	33 (65%)	Ref
Other (incl. pub/restaurant/others' home)	18 (35%)	3.33 0.98, 11.29
Company		
Family	19 (30%)	Ref
Friends	32 (51%)	7.5 (1.29, 43.33)*
Combined family/friends	9 (14%)	6.8 (0.85, 54.36)
Other	3 (5%)	4.3 (0.27, 67.44)
Mean no. of people watching the game with	3 (range 0–14)	
Less than 3 people	32 (52%)	Ref
3 or more people	30 (48%)	1.39 (0.40, 4.85)
Gender of company^b^		
Men	25 (41%)	Ref
Women	13 (21%)	1.09 (0.03, 1.25)
Mixed group	22 (36%)	0.51 (0.16, 1.57)
Age of company^b^		
Under 30	31 (51%)	Ref
Over 30	14 (23%)	0.73 (0.13, 4.11)
Mixed	15 (25%)	1.21 (0.33, 4.42)

Abbreviations: AUDIT‐C, Alcohol Use Disorders Identification Test; CI, confidence interval; OR, odds ratio.

^a^ No participants identified as non‐binary.

^b^ A participant watched one game alone.

* *p* < 0.05.

Results from the binary logistic regression analyses are also shown in Table 1. RSOD was more likely for early‐afternoon games (starting between 1 and 3 pm) rather than late‐afternoon games (starting between 3 and 6 pm). RSOD was also more likely if participants viewed the game at a stadium or pub (compared to home), and if they were with friends (compared to family).

**FIGURE 1:**
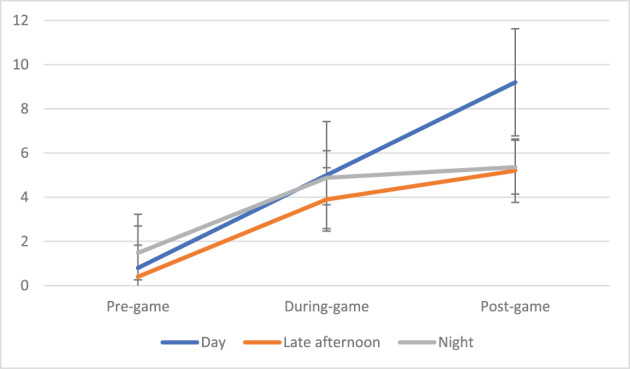
Mean drinks before, during and after the game, by time of match.

**FIGURE 2:**
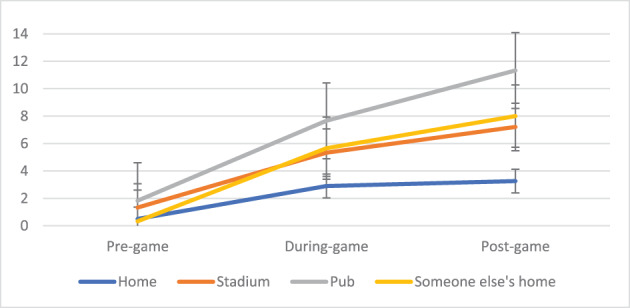
Mean drinks before, during and after the game, by location of match viewing.

**FIGURE 3:**
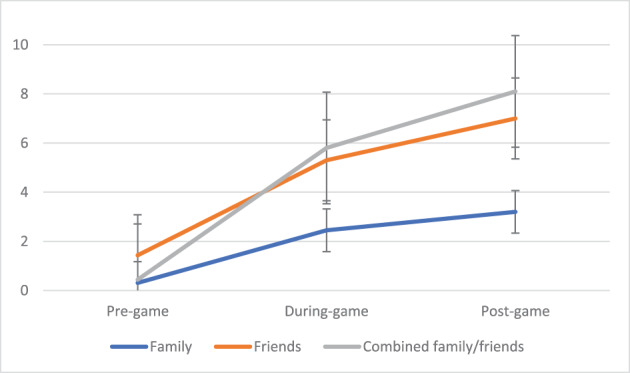
Mean drinks before, during and after the game, by company of match viewing.

Figures 1, 2, 3 present the cumulative mean sample drinks at pre‐game, during‐game and post‐game, of those findings significant in the regression analyses. Figure 1 shows that pre‐game and during‐game drinking were similar regardless of game time; but post‐game drinking was much greater after early‐afternoon games. Pairwise comparisons indicated that pre‐drinking was higher before night games (compared to late‐afternoon games) (1.5 vs. 0.3 drinks, *t* = 2.88, *F*(17.2, 99.6) = 8.29, *p* = 0.015, Cohen's *d* = 0.82) and post‐game drinking higher after early‐afternoon games (compared to night games) (3.5 vs. 0.4 drinks, *t* = 3.01, *F*(84.0, 381.9) = 8.36, *p* = 0.011, Cohen's *d* = 0.97).

Figure 2 shows that alcohol consumption was higher for those watching the game at a pub compared to home. Pairwise comparisons indicated that this was due to more drinks during the game for those at the pub (5.8 vs. 2.1 drinks, *t* = 2.91, *F*(70.2, 143.8) = 14.6, *p* = 0.04, Cohen's *d* = 1.7) as well as in total (11.3 drinks vs. 3.3 drinks, *t* = 3.54, *F*(357.5, 603.9) = 17.7, *p* = 0.007, Cohen's *d* = 1.9). Figure 3 shows that drinking was higher when participants watched the game with friends or a combination of friends and family (compared to just family). Pairwise comparisons detected a significant difference in drinks consumed during the match when watching with a combination of family and friends compared to family alone (5.4 vs. 1.8 drinks, *F*(70.2, 143.8) = 14.6, *t* = 3.07, *p* = 0.017, Cohen's *d* = 0.55).

## 4 DISCUSSION

The findings of this pilot study show EMA to be a feasible and useful data collection method for understanding drinking behaviours before, during and after viewing professional sporting events. The primary feasibility problem we encountered was in relation to recruitment (covered in more detail in the Section 4.1). However, once engaged in the study, participants responded well to survey prompts (85.5% completion rate) and, despite our sample size limitations, we were able to detect some significant effects for social and settings‐based characteristics that require replication in larger studies.

Our sample reported consuming an average of six drinks across the match (pre, during and post). This is consistent with another Victorian study where patrons reported consuming six standard drinks at sports bars [8]. Late afternoon games involved less drinking (particularly post‐drinking) than early afternoon games. This shows scheduling to be an important predictor of consumption, a characteristic also evident with American football [4]. This finding also suggests that the earlier the game, the earlier spectators start drinking, often continuing to drink afterwards. These findings are consistent with a study of young adults in the Netherlands, which showed that drinkers do not ‘catch up’ on missed drinks if they begin drinking later [17]. If this is confirmed in a larger study, it might be a useful consideration for match scheduling. For example, marquee games with large crowds and high‐profile teams where more alcohol‐related problems are expected (e.g., [7]) might be scheduled in the late afternoon to reduce heavy drinking. However, it is unknown in this context whether heavier drinking equates to greater experience of alcohol‐related negative consequences. Indeed, moving matches later may increase the amount of people in venues later, which may increase consequences regardless of lower consumption. Future research on this topic should investigate both consumption and consequences (i.e., using the next day survey to ask about consequences).

Social and environmental characteristics were found to be important, as identified in previous event‐level studies [9, 10, 11]. RSOD was more likely if participants watched the game at a stadium or pub compared to their own home. This highlights the importance of promoting safe drinking practices and policies (e.g., responsible service of alcohol) at sports stadiums and licensed venues. RSOD was also more likely if participants watched the game with friends or a combination of friends and family (compared to family alone). We did not identify a relationship between group size and RSOD, nor for the gender and age of the participants' company. This is different to event‐level studies among young patrons in nightlife areas [9, 10, 11], but is aligned with the limited event‐level research undertaken at sports events [4]. While needing replication in larger studies, it may be that women and older spectators are not protective for drinking before, during and after sporting events in the same way as they are in nightlife environments. The strong limiting effect on alcohol consumption of watching with only family suggests that promoting the AFL as a family environment might be a useful avenue for consideration, as is evident in non‐professional football contexts [18].

Pre‐drinking was more likely before night games, and post‐drinking was more likely after day games. While this finding is not surprising, it does underscore the importance of alcohol and patron safety policies. For example, stadium managers need to emphasise the responsible service of alcohol and ensure appropriate security for night games given patrons are more likely to have consumed alcohol prior to entering the stadium. This is also an important post‐game consideration for licensed venues in close proximity to stadiums or that are broadcasting AFL games. Unfortunately, we did not ask participants where they had consumed alcohol before the game, this information will be informative in future research.

### 4.1 Limitations and considerations for future research

Pilot study sample size limitations mean findings should be interpreted cautiously. We deliberately recruited people who attend AFL games and consume alcohol regularly to understand the practices of those ‘at risk’ and our findings are not indicative of general drinking patterns while watching AFL games. We initially recruited participants outside an AFL stadium because we wanted to target sports fans who attend matches at stadiums at least sometimes (to evaluate the importance of location of viewing), but this was resource intensive and most participants who were screened did not agree to participate in the EMA study when contacted later. In future research we might consider working with AFL teams to test the viability of recruiting through newsletters to club members, in addition to social media recruitment.

We also had a sizeable amount of missing data (14.5% of surveys). However, because survey participants were asked how much alcohol they had consumed since their last survey, we were still able to capture total consumption. Missing data, however, are not ideal, given the potential for recall bias, and the significant social, contextual, mood and practice‐based changes that can occur quickly during a sporting event. Due to the pilot nature of the study, we only collected data from participants for one match each weekend—a match that they pre‐selected—and it is likely that this is a match for which they planned to attend or drink alcohol. However, it is probable that participants watched more matches than one per weekend and perhaps engaged in unplanned drinking while watching other matches. Using a more flexible data collection approach, where participants do not have to rely on the initial trigger surveys, in addition to inputting data without the survey notifications (i.e., a diary approach), might be useful in larger studies. It is also important to note that we did not collect data on respondents' drinking during non‐AFL watching weekends, which means it is not clear whether respondents drink differently when watching football compared to other weekend activities. Regardless, the risks of heavy drinking for AFL spectators remain a key concern and our data point to some potential pathways for intervention and research.

We were underpowered to explore the role of participant gender and age in this study, but both have been identified as important in international event‐level research [4, 8, 9], and how demographics shape RSOD during AFL matches requires further investigation in a larger sample. While expectations about the outcome of the match, and actual outcome of the match did not predict RSOD, a closer examination of mood might be useful given its association with drinking in other event‐level research [9]. Further investigation of how crowd size and match profile (e.g., marquee games) influence drinking is also needed. A larger sample size will enable an examination of event‐level sequences and how shifts in a particular factor might shape drinking (e.g., context, company and mood). More detail on adverse outcomes from alcohol use is also needed, such as whether involvement in alcohol‐related altercations or accidents occur during the event. It is possible, for example, that drinking may be heavier during the day, but rates of harm are greater at night.

## 5 CONCLUSIONS

Findings from this pilot study endorse the feasibility of EMA research with Australian sports fans. Preliminary findings suggest that social and contextual factors impact how alcohol is consumed in association with AFL games. These findings require further investigation in larger samples, so as to meaningfully inform prevention and policies to reduce the rates of harm that occur after AFL matches [7].

## AUTHOR CONTRIBUTIONS

Each author certifies that their contribution to this work meets the standards of the International Committee of Medical Journal Editors. All authors were substantively involved in research design. Kelly van Egmond, Dan Anderson‐Luxford, Cassandra J. C. Wright and Gabriel Caluzzi collected data. Kelly van Egmond and Dan Anderson‐Luxford prepared data for analysis. Amy Pennay and Emmanuel Kuntsche led analyses. Amy Pennay led manuscript writing with all authors contributing to editing. All authors have approved the final version of the manuscript.

## CONFLICT OF INTEREST STATEMENT

The authors declare no conflict of interest.
